# Requirement of a Wnt5A–microbiota axis in the maintenance of gut B-cell repertoire and protection from infection

**DOI:** 10.1128/msphere.00204-24

**Published:** 2024-08-14

**Authors:** Soham Sengupta, Malini Sen

**Affiliations:** 1 CSIR-Indian Institute of Chemical Biology, Kolkata, West Bengal, India; 2 Bio Bharati Life Science Pvt. Ltd., Kolkata, West Bengal, India; University of Michigan-Ann Arbor, Ann Arbor, Michigan, USA

**Keywords:** Wnt5A, commensal microbiota, B cell, IgG1, IgA, IgM, *Salmonella typhimurium*

## Abstract

We investigated the influence of a Wnt5A–gut microbiota axis on gut B-cell repertoire and protection from infection, having previously demonstrated that Wnt5A in association with gut commensals helps shape gut T-cell repertoire. Accordingly, Wnt5A heterozygous mice, which express less than wild-type level of Wnt5A, and their isolated Peyer’s patches (PPs) were studied in comparison with the wild-type counterparts. The percentages of IgM- and IgA-expressing B cells were quite similar in the PP of both sets of mice. However, the PP of the Wnt5A heterozygous mice harbored significantly higher than wild-type levels of microbiota-bound B cell-secreted IgA, indicating the prevalence of a microbial population therein, which is significantly altered from that of wild-type. Additionally, the percentage of PP IgG1-expressing B cells was appreciably depressed in the Wnt5A heterozygous mice in comparison to wild-type. Wnt5A heterozygous mice, furthermore, exhibited notably higher than the wild-type levels of morbidity and mortality following infection with *Salmonella typhimurium*, a common gut pathogen. Differences in morbidity/mortality correlated with considerable disparity between the PP–B-cell repertoires of the *Salmonella*-infected Wnt5A heterozygous and wild-type mice, in which the percentage of IgG1-expressing B1b cells in the PP of heterozygous mice remains significantly low as compared to wild-type. Overall, these results suggest that a gut Wnt5A–microbiota axis is intrinsically associated with the maintenance of gut B-cell repertoire and protection from infection.

**IMPORTANCE:**

Although it is well accepted that B cells and microbiota are required for protection from infection and preservation of gut health, a lot remains unknown about how the optimum B-cell repertoire and microbiota are maintained in the gut. The importance of this study lies in the fact that it unveils a potential role of a growth factor termed Wnt5A in the safeguarding of the gut B-cell population and microbiota, thereby protecting the gut from the deleterious effect of infections by common pathogens. Documentation of the involvement of a Wnt5A–microbiota axis in the shaping of a protective gut B-cell repertoire, furthermore, opens up new avenues of investigations for understanding gut disorders related to microbial dysbiosis and B-cell homeostasis that, till date, are considered incurable.

## OBSERVATION

Gut microbiota and the immune network remain closely linked in the fight of the human host against infection by common pathogens (1, 2). Yet, the basis of association of gut microbiota with immune cells in the context of immune defense remains unexplained. We previously demonstrated that Wnt5A in connection with gut commensals helps shape the gut immune T-cell repertoire at the steady state (3). In view of the functional interdependence of B and T cells (4–6) and the influence of gut microbiota on B-cell differentiation (7), we presently explored the possibility of an association of a Wnt5A–gut commensal axis with the shaping of gut B-cell repertoire and thereby protection from infection. This investigation was further prompted by experimental findings indicating gut commensals as a potential regulator of antibody class switching, a major aspect in the development and maintenance of B-cell repertoire (8–10). Our findings reveal that a Wnt5A–gut commensal axis indeed influences the gut B-cell repertoire and resistance of the host toward infection.

### Wnt5A dosage-linked alteration in gut commensals affects the gut B-cell repertoire

**Fig 1:**
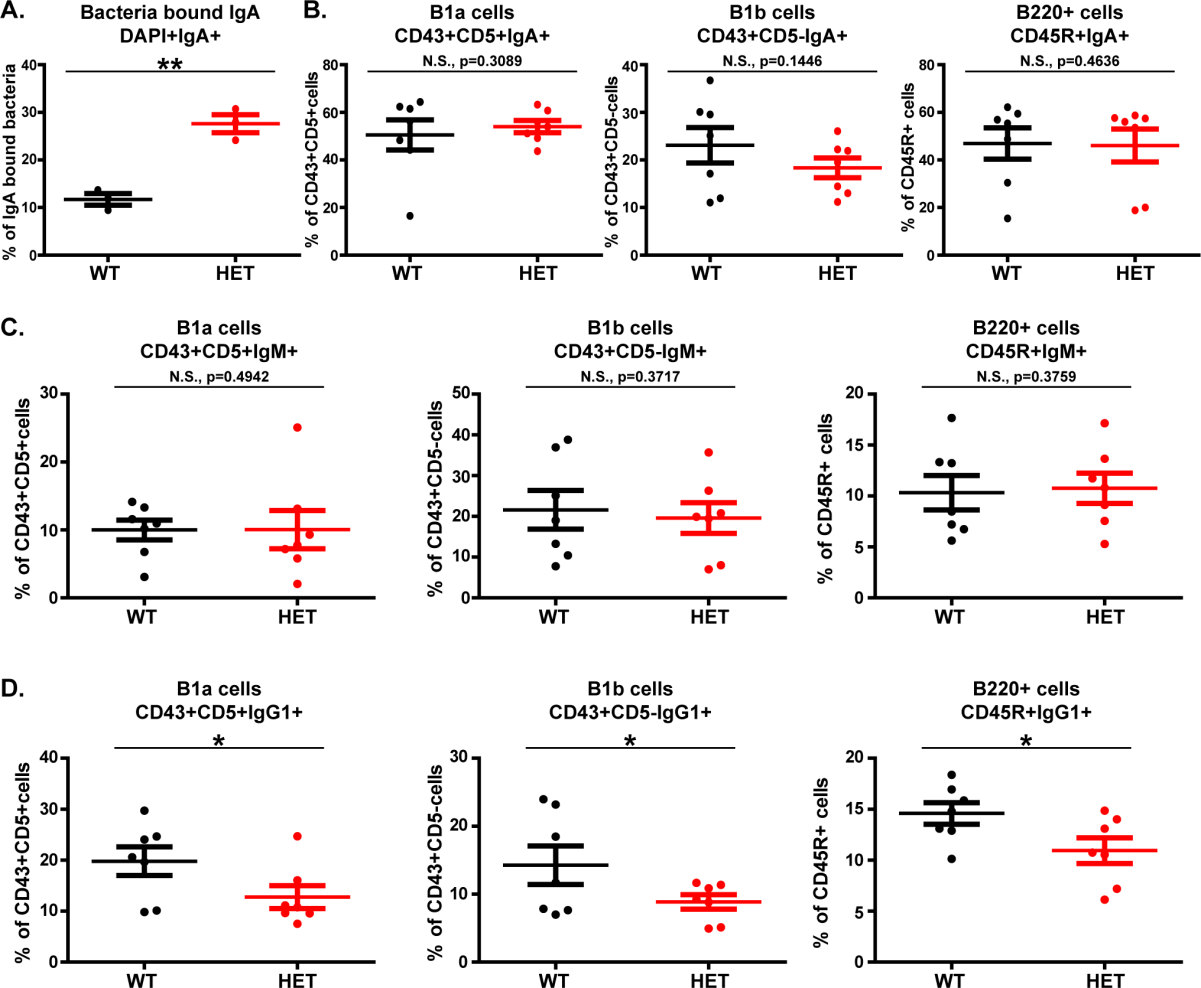
Wnt5A dosage controls the steady-state level of gut (PP) sIgA-bound bacteria and B-cell repertoire. (**A**) Graph representing increased percentage of bacteria-bound sIgA in the PP of Wnt5A heterozygous (HET) mice in comparison to wild-type (WT) (*n* = 3). (B and C) There is no significant change in the percentage of IgA-expressing (**B**) and IgM-expressing (**C**) B1a (CD43^+^ and CD5^+^), B1b (CD43^+^ and CD5^−^) and CD45R/B220^+^ B cells between the Wnt5A heterozygous and wild-type mice, as represented graphically (*n* = 7). (**D**) Plots showing significantly lower percentage of IgG1-expressing B1a, B1b, and B220^+^ B cells in the PP of Wnt5A heterozygous mice as compared to wild-type (*n* = 7). Data represented as mean ± SEM. *P* ≤ 0.05 was considered as significant statistically. Significance is represented by * in the following manner: *P ≤ 0.05, **P ≤ 0.01, ***P ≤ 0.001. “N.S.” denotes non-significant. “n” represents number of mice in each experimental set.

In order to decipher if a Wnt5A–gut commensal axis influences gut B-cell repertoire, we compared wild-type mice with Wnt5A heterozygous mice (harboring one functional copy of the Wnt5A gene), where reduced Wnt5A expression with reference to wild-type correlates with altered abundance of the gut microbiota and a potential for dysbiosis (3). In both Wnt5A heterozygous and wild-type mice, we quantified IgM/IgA/IgG1-expressing B-cell subtypes (B220^+^: bone marrow-derived mature B cells, and B1a/B1b: independent B-cell lineage important for innate immunity) in the respective Peyer’s patches (PPs). Both IgM- and IgA-expressing B cells were targeted due to the abundance of IgM and IgA in the gut (11–13). IgG1-expressing B cells were targeted on account of reports demonstrating IgG1-mediated immune protection of the gut against infection by *Salmonella*, a common gut pathogen (9, 14). Alongside, we measured bacteria-bound IgA in the PP of all sets of mice in line with its strong correlation with alteration of microbial abundance and dysbiosis, as well as prevalence of gut infection (4, 15–20). Experiments performed in the steady state revealed significantly higher level of bacteria-bound secreted IgA (sIgA) but similar percentages of IgM- and IgA-expressing B220^+^, B1a, and B1b cells in the PP of the Wnt5A heterozygous mice as compared to the wild-type counterparts (Fig. 1A to C). The high bacteria-bound sIgA level in the Wnt5A heterozygous mice validated alteration in gut bacterial abundance therein as compared to the wild-type, in line with our previously published study where alteration in bacterial abundance in the Wnt5A heterozygous mice was verified by 16S sequencing (3, 16, 18–20). In conjunction with increase in bacteria-bound sIgA, there was significantly depressed percentage of PP IgG1-expressing B cells (B1a, B1b, and B220) in the Wnt5A heterozygous mice in comparison to wild-type (Fig. 1D). The observed difference between Wnt5A heterozygous and wild-type mice in B-cell IgG1 expression could be due to variation in B-cell class switching caused by altered abundance in gut microbiota (7–10). All gating of flow cytometry pertaining to Fig. 1 is demonstrated in Fig. S1. These results suggest that a Wnt5A dosage–gut commensal axis contributes to shaping gut B-cell repertoire.

### A Wnt5A–gut commensal axis linked B-cell repertoire correlates with protection from damage caused by infection

**Fig 2:**
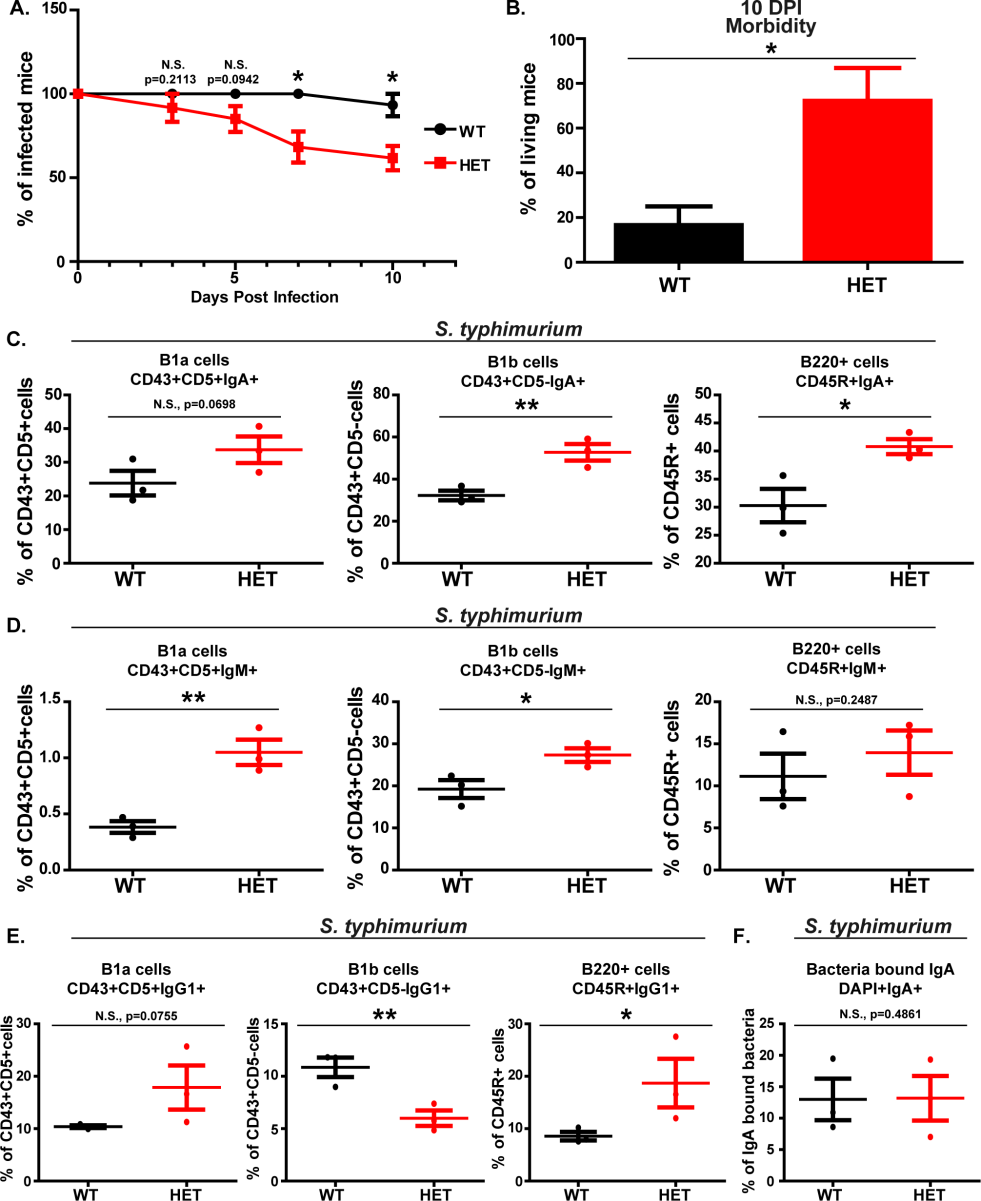
Wnt5A dosage-dependent morbidity/mortality after *Salmonella* infection correlates with an altered B-cell repertoire. (**A**) Survival curve showing depletion in percentage of healthy Wnt5A heterozygous (HET) mice as compared to the wild-type (WT) controls, due to significant increase in mortality under *S. typhimurium*-infected conditions, as noted 3, 5, 7, and 10 days post-infection (DPI) (*n* = 3 separate experiments with four to five mice in each WT/HET group). (**B**) Graph demonstrating the percentage of live mice that were morbid at 10 days post-infection. Mice were marked as morbid, as per their scores explained in Table 1. (C and D) Graphs depicting higher percentage of IgA (**C**) and IgM (**D**) expressing CD45R/B220^+^, B1a (CD43^+^ and CD5^+^), and B1b (CD43^+^ and CD5^−^) cells in the PP of *S. typhimurium*-infected Wnt5A heterozygous mice in comparison to the wild-type counterparts at 10 DPI. The increase in B1a cell IgA and B220 cell IgM however was not statistically significant (*n* = 3). (**E**) Graphs demonstrating that *S. typhimurium*-infected Wnt5A heterozygous mice PP had higher percentage of IgG1-B220^+^ and IgG1-B1a cells (although not statistically significant) but significantly lower percentage of IgG1-B1b cells as opposed to the wild-type controls at 10 DPI (*n* = 3). (**F**) Plot depicting no difference between sIgA-bound bacteria in the PP of *S. typhimurium*-infected wild-type and heterozygous mice. Data represented as mean ± SEM. *P* ≤ 0.05 was considered as significant statistically. Significance was represented by * in the following manner: *P ≤ 0.05, **P ≤ 0.01, ***P ≤ 0.001. “N.S.” denotes non-significant. “n” represents number of mice in each experimental set.

Wnt5A wild-type and heterozygous mice were afresh used to study how the Wnt5A dosage–gut commensal axis-associated B-cell repertoire correlates with infection. In view of the already documented increased association of potential pathogens such as *Helicobacter* sp. and *Prevotella* sp. with the PP of Wnt5A heterozygous mice as compared to the wild-type (3), as well as the propensity of sIgA binding to potential pathogens (4, 16, 18–20), the observed difference between the Wnt5A wild-type and heterozygous mice in bacteria-bound sIgA (Fig. 1) suggested higher probability of gut infection-associated damage in the Wnt5A heterozygous mice as opposed to the wild-type. The already documented IgG1-dependent protection against *Salmonella* infection (9, 14) and the low level of IgG1-expressing B cells in the Wnt5A heterozygous mice as compared to wild-type (Fig. 1), furthermore, strengthened the possibility of increased damage from *Salmonella* infection in the Wnt5A heterozygous mice. Accordingly, we checked the outcome of *Salmonella typhimurium* infection in the Wnt5A heterozygous and wild-type mice. Both sets of mice were infected to the same extent through oral gavage (~10^7^ *S. typhimurium* per mouse). The infection load in the PP and spleen 10 days post-infection was moderately higher in the heterozygous mice as compared to wild-type (Fig. S2). In line with depressed IgG1-expressing B cells in the Wnt5A heterozygous mice at steady state, the infected heterozygous mice were significantly more morbid than the wild-type counterparts as explained in Table 1; Fig. 2 indicates greater damage on account of infection. Morbidity was scored based on posture, movement, hunger and thirst, grooming, respiration, state of fur, and interaction with the environment. High morbidity scores correlated with mortality as evident from percent survival (Fig. 2A and B). High morbidity/mortality in the Wnt5A heterozygous mice was associated with higher than wild-type percentages of PP-associated IgA/IgM/IgG1-expressing B-cell subtypes (higher IgA and IgM: B1a, B1b, and B220; higher IgG1: B1a and B220) (Fig. 2C to E), which could act as drivers of inflammation (21–24). On the other hand, the percentage of IgG1-expressing B1b cells, which are important for warding off bacterial infections along with B1a cells (25–30), remained much lower in the Wnt5A heterozygous mice than in the wild-type (Fig. 2E). The extent of sIgA-bound bacteria in both wild-type and Wnt5A heterozygous mice was the same (Fig. 2F) despite the presence of increased IgA-expressing B cells in the heterozygous, suggesting lack of sIgA-mediated suppression of infection through bacterial binding. All gating of Flow Cytometry pertaining to Fig. 2 is explained in Fig. S3. These observations suggest that a Wnt5A dosage–gut commensal axis-linked B-cell repertoire is important for protection from the deleterious effects of infection.

**TABLE 1:** Morbidity scoring of *S. typhimurium*-infected mice^*a*^

Mouse type	Appetite and thirst	Respiration	Movement	Interaction with environment	Grooming	Posture	Cage organization	Fur	Reaction to stimulus	Eyes	Total	Status at day 10
Set 1												
WT1	0	0	0	0	0	1	0	0	1	0	2/10	H
WT2	1	0	1	1	1	1	1	0	1	1	8/10	M
WT3	1	0	0	0	0	0	0	1	0	1	3/10	H
WT4	0	0	0	1	0	0	0	0	1	0	2/10	H
HET1	1	1	1	1	1	1	1	1	1	1	10/10	M
HET2	1	1	1	1	1	1	0	1	1	1	9/10	M
HET3	Died at day 3											D
HET4	Died at day 6											D
Set 2												
WT5	1	1	1	1	1	1	1	1	1	1	10/10	M
WT6	1	0	0	0	0	0	0	0	0	0	1/10	H
WT7	0	0	0	0	0	0	0	0	0	0	0/10	H
WT8	0	0	1	1	0	0	0	0	1	0	3/10	H
HET5	1	1	1	1	1	1	1	1	1	1	10/10	M
HET6	1	1	1	1	1	1	1	1	1	1	10/10	M
HET7	Died at day 7											D
HET8	1	1	1	1	1	1	1	1	1	0	9/10	M
Set 3												
WT9	0	0	0	0	0	0	0	0	1	1	2/10	H
WT10	0	0	0	0	0	0	1	0	0	0	1/10	H
WT11	Died at day 9											D
WT12	0	0	0	0	0	0	0	0	0	0	0/10	H
WT13	1	0	1	1	0	0	0	0	1	0	4/10	H
HET9	Died at day 4											D
HET 10	1	0	1	1	0	0	0	0	1	0	4/10	H
HET 11	1	1	1	1	1	1	1	1	1	0	9/10	M
HET 12	1	1	1	1	1	1	1	0	1	0	8/10	M
HET 13	Died at day 8											D

^*a*^ D, dead; H, healthy; HET, Wnt5A heterozygous mouse; M, morbid; WT, wild-type mouse.

### Shaping of the gut B-cell repertoire

Our results indicate that the gut microbiota abundance/profile that is dependent on the dosage of Wnt5A is associated with shaping of the gut B-cell repertoire. Whether the effect of Wnt5A dosage–gut microbiota axis on the B cells is T cell-dependent, independent, or a combination of both is unclear at this stage and warrants further investigation. Nevertheless, it is clear that alterations in the gut microbial population due to compromised levels of Wnt5A influence the gut B-cell repertoire. Such alterations could promote damage from infection and associated inflammation, as often is the case during microbial dysbiosis (17, 20, 31–35). The high level of morbidity/mortality in the infected Wnt5A heterozygous mice as compared to the wild-type is quite likely due to the weakened ability to combat pathogenic conditions associated with infection.

The potential role of the Wnt5A–gut microbiota axis in the maintenance of gut B-cell repertoire and protection from the harmful effects of infection, as projected through this study, opens up new avenues for further investigations into the role of Wnt5A signaling in gut health and disease.

### Reagents

All reagents used in this study are enlisted in Table 2.

**TABLE 2:** All reagents used in this study are enlisted below

Reagent or resource	Source	Ref. no.
RPMI 1640	Life Technologies (Thermo Fisher Scientific, USA)	31800-022
HI FBS	Life Technologies (Thermo Fisher Scientific, USA)	10082147
L-glutamine	Life Technologies (Thermo Fisher Scientific, USA)	25030081
DAPI	Life Technologies (Thermo Fisher Scientific, USA)	D1306
DNase I	Life Technologies (Thermo Fisher Scientific, USA)	AM2222
Collagenase D	Sigma-Aldrich, USA	11088866001
NaCl	Sigma-Aldrich, USA	S5886
Fixable Viability Stain 450	BD Biosciences, USA	562247
APC rat anti-mouse CD5	BD Biosciences, USA	550035
PeCy7 rat anti-mouse CD43	BD Biosciences, USA	562866
PeCy7 rat anti-mouse CD45R/B220	BD Biosciences, USA	561881
PerCP Cy5.5 rat anti-mouse IgM	BD Biosciences, USA	550881
BV605 rat anti-mouse IgG1	BD Biosciences, USA	563285
FITC rat anti-mouse IgA	BD Biosciences, USA	559354

### Mice maintenance, PP isolation, and infection

Wnt5A wild-type (+/+) and heterozygous (+/−) mice belonging to the strain B6;129S7-*Wnt5a^tm1Amc^/*J obtained from Jackson Laboratory were bred and maintained in the institutional animal house facility of CSIR-IICB as previously described (3). Mice, 8- to 10-week-old, used for control and experimental sets, had similar male:female ratio.

Cells from PPs were isolated following previously published protocol (3). Briefly, the PPs identified from the small intestine were isolated as 1-mm sections, shaken for 20 min at 37°C with 200 revolutions per minute in RPMI 1640 media containing 10% fetal bovine serum (FBS) and 10 mM EDTA, following which media was changed with RPMI 1640 (+10% FBS) containing collagenase D and DNase I for further 15-min shaking under similar conditions. After repeated dispersion by passage through 40-µm cell strainer and short-term shakings, the almost dispersed single cells were used for experiments.

Mice were infected with *Salmonella enterica* subsp. *enterica* serovar Typhimurium, also known as *S. typhimurium* purchased from MTCC, Chandigarh, India (MTCC 3224), at dosage of 10^7^ bacteria per mouse via oral gavage, following previously published protocols (36, 37).

### Flow cytometry

Cells were prepared for flow cytometry following previously published protocols (3). Briefly, PP cells were incubated in 1% bovine serum albumin (BSA:blocking agent) and then incubated at 4°C with fluorophore-conjugated antibodies dissolved in 0.5% BSA in phosphate-buffered saline (PBS) for 1 h. B-cell subset identification was done with appropriate antibodies using flow cytometry.

For studying bacteria-bound sIgA, PP cells were maintained overnight in antibiotic-free RPMI 1640 medium supplemented with 10% FBS and 2 mM L-glutamine. The harvested sup was first centrifuged for 5 min at 700 *g* to remove floating eukaryotic cells, and centrifuged again at 7,155 × *g* for 3 min to pellet down bacterial cells. The pellet was suspended in 0.5% BSA and PBS and stained with fluorophore-bound anti-IgA antibody, following counterstaining with 4′,6-diamidino-2-phenylindole (DAPI) (bacteria identification). Data acquisition was done using BD.LSR Fortessa Cell Analyzer, and flow cytometric data analysis was done by FCS Express 5 software. All cell percentages are calculated and presented on the basis of the previous gate.

### Scoring of morbidity in *S. typhimurium*-infected mice

Mice infected with *Salmonella* sp. at 10^7^ were evaluated for morbidity 10 days post-infection, by using a modified version of a previously published strategies (38, 39). Morbidity was scored on a scale of 10, by considering 10 different factors. For the factors, appetite and thirst, movement, interaction with environment, grooming, cage organization, and reaction to stimulus, 0 was considered as normal, and 1 was considered as indication of disease. This was the case also for evaluation of respiration, fur, posture, and eyes. In the case of respiration, 0 was considered normal breathing, and 1 was considered heavy breathing, indicating disease. For fur, 0 was normal, and 1 was rough and was considered diseased. For posture, 0 was normal and 1 was considered hunched (disease). In the case of eyes, 0 marked normal eyes, and 1 indicated drowsy (disease) eyes. Mice getting scores above 5 were considered morbid.

### RT qPCR

Reverse transcription quantitative PCR (qPCR) was conducted by isolating RNA from PPs and spleen, following previously published protocol (3). The primers used for identification of *Salmonella* sp. were forward 5′ ACTGGAAACGGTGGCTAATAC 3′ and reverse 5′ CTCACCAACTAGCTAATCCCATC 3′. Internal control glyceraldehyde 3-phosphate dehydrogenase (GAPDH) was measured using 5′ AACAGCAACTCCCACTCTTC 3′ as forward and 5′ CCTGTTGCTGTAGCCGTATT 3′ as reverse primers. Fold change in bacterial load (2^−ΔΔCt^) was calculated as demonstrated in Fig. S2, following previously published formulae (40).

### Statistical analysis

Statistical analysis was done utilizing paired or unpaired Student’s *t* test as per need using GraphPad Prism 5 software. Graphs and line diagrams are represented as mean ± SEM, and *P* ≤ 0.05 was considered statistically significant. Significance was represented by * in the following manner: *P ≤ 0.05, **P ≤ 0.01, and ***P ≤ 0.001.

## SUPPLEMENTAL MATERIAL

Figures S1 to S3 msphere.00204-24-s0001.pdf Flow cytometry gating strategies and enumeration of bacterial infection.

ASM does not own the copyrights to Supplemental Material that may be linked to, or accessed through, an article. The authors have granted ASM a non-exclusive, world-wide license to publish the Supplemental Material files. Please contact the corresponding author directly for reuse.
