# Supplementary material for: Requirement of a Wnt5A–microbiota axis in the maintenance of gut B-cell repertoire and protection from infection
Source: mSphere. 2024 Aug 14;9(9):e00204-24. doi: 10.1128/msphere.00204-24 (PMC11423572; doi:10.1128/msphere.00204-24)
Supplement: Figures S1 to S3 — Flow cytometry gating strategies and enumeration of bacterial infection. [file msphere.00204-24-s0001.pdf]

Supplementary Fig. 1

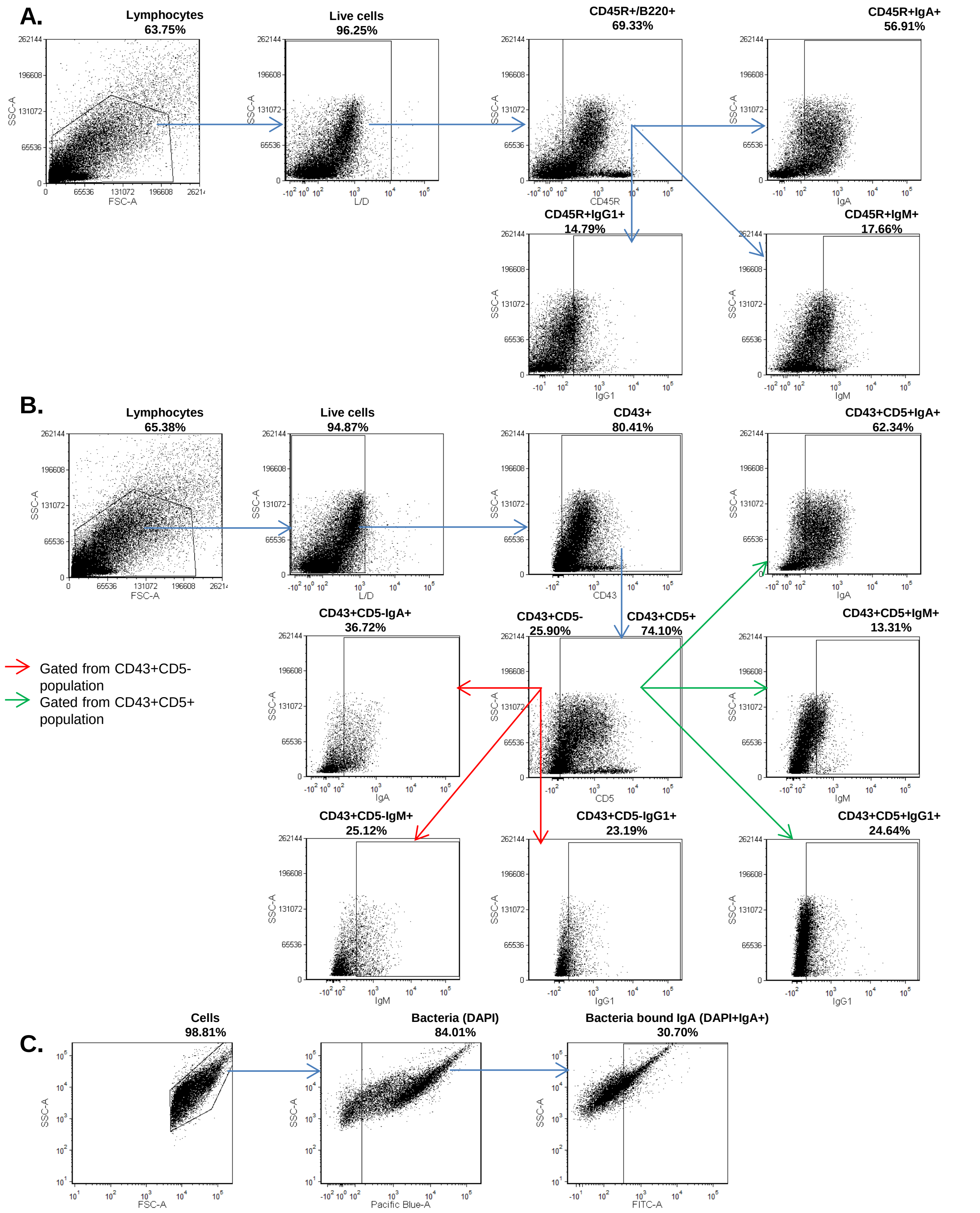

Supplementary Figure 1: Gating strategy to evaluate Immunoglobulin expression in B cell subsets, and sIgA bound bacteria in PP at the steady state. (A) Gating strategy used to study IgA, IgM and IgG1 expression in CD45R+/B220+ B cell population. (B) Gating strategy used to study IgA, IgM and IgG1 expression in B1a (CD43+CD5+) and B1b (CD43+CD5-) cell populations. (C) Gating strategy used to study bacteria bound sIgA. Percentage mentioned on top of each plot is the percent of positive cells of the previous gate.

Supplementary Fig. 2

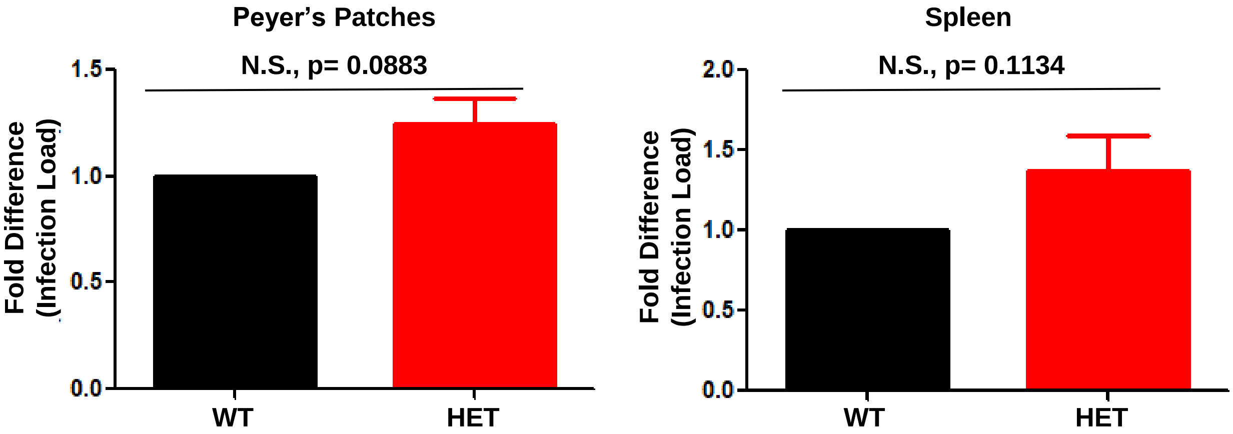

Calculation was done as below:
ΔC_t= (C_t of *Salmonella sp.* specific 16S region – C_t of GAPDH internal control).
$\Delta\Delta C_t$= (ΔC_t of Heterozygous mouse - ΔC_t of Wild Type mouse).
$2^{-\Delta\Delta Ct}$ = Fold difference of genetic material (Infection load) between Heterozygous and wild type mice.

Supplementary Figure 2: Enumeration of the load of *S. typhimurium* in Spleen and Peyer's patches of mice at 10 days post infection. Graphs demonstrating the fold difference ($2^{-\Delta\Delta Ct}$) in infection load between Wnt5A heterozygous (HET) and wild type (WT) mice at 10 DPI (Days Post Infection) in Spleen and Peyer's patches (PP). Fold difference is plotted considering infection load of WT mice as 1. Formula used for calculation of fold change ($2^{-\Delta\Delta Ct}$) in infection is documented in Schmittgen et al. (Nature Protocol, 2008). (n=3). Data represented as mean ± SEM. $p \leq 0.05$ was considered as significant statistically. Significance was represented by * in the following manner: * $p \leq 0.05$, ** $p \leq 0.01$, *** $p \leq 0.001$. "N.S." denotes non-significant. "n" represents the number of mice in each set.

Supplementary Fig. 3

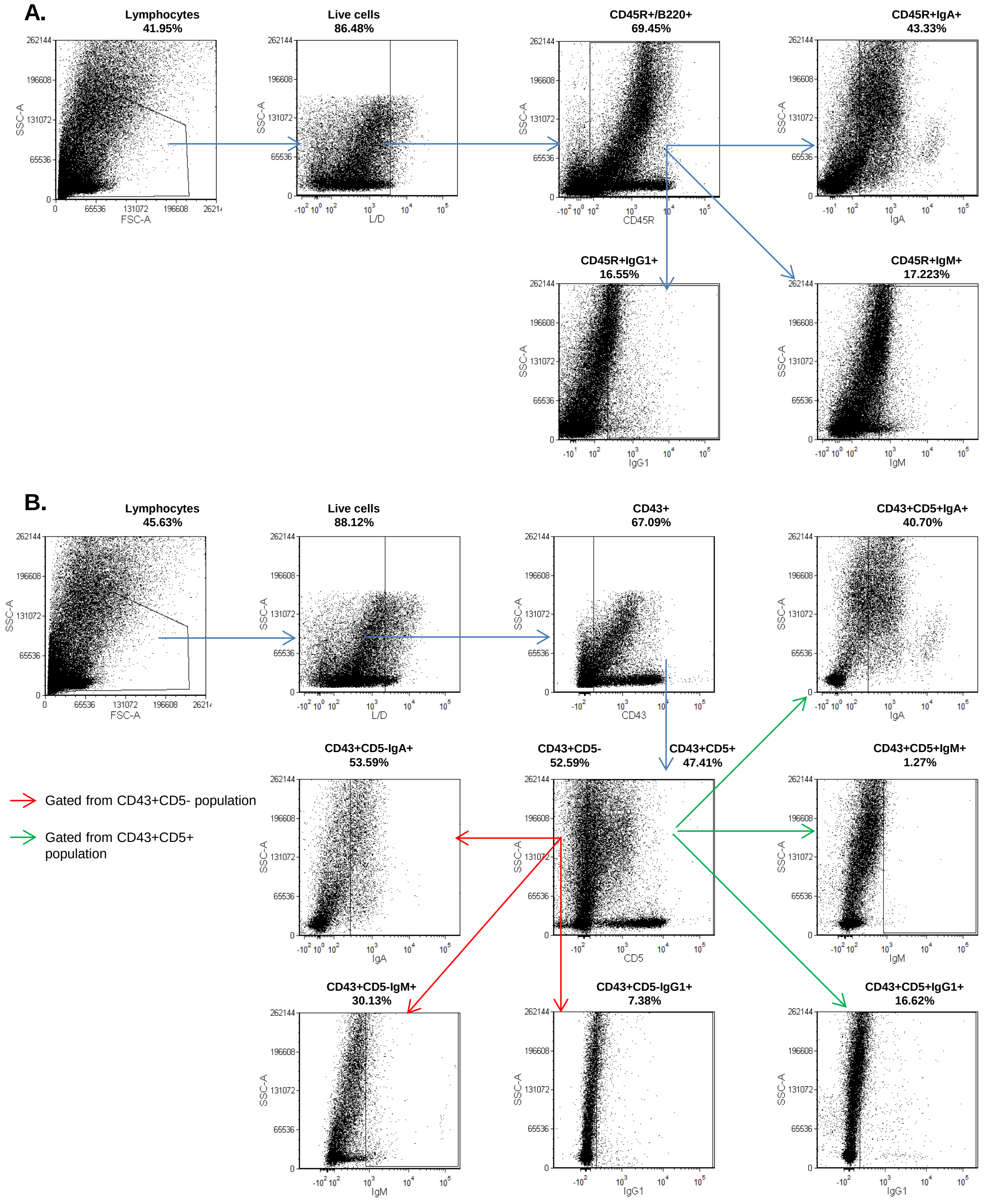

Supplementary Figure 3: Gating strategy to study Immunoglobulin expression in the PP of *S. typhimurium* infected mice. (A) Gating strategy used to study IgA, IgM and IgG1 expression in CD45R+/B220+ B cell population. (B) Gating strategy used to study IgA, IgM and IgG1 expression in B1a (CD43+CD5+) and B1b (CD43+CD5-) cell populations.
